# Air Pollution and Mortality in Seven Million Adults: The Dutch Environmental Longitudinal Study (DUELS)

**DOI:** 10.1289/ehp.1408254

**Published:** 2015-03-11

**Authors:** Paul H. Fischer, Marten Marra, Caroline B. Ameling, Gerard Hoek, Rob Beelen, Kees de Hoogh, Oscar Breugelmans, Hanneke Kruize, Nicole A.H. Janssen, Danny Houthuijs

**Affiliations:** 1 National Institute for Public Health and the Environment, Bilthoven, the Netherlands; 2 Institute for Risk Assessment Sciences, Utrecht University, Utrecht, the Netherlands; 3 MRC-PHE Centre for Environment and Health, Department of Epidemiology and Biostatistics, Imperial College London, London, United Kingdom; 4 Swiss Tropical and Public Health Institute, Basel, Switzerland; 5 University of Basel, Basel, Switzerland

## Abstract

**Background:**

Long-term exposure to air pollution has been associated with mortality in urban cohort studies. Few studies have investigated this association in large-scale population registries, including non-urban populations.

**Objectives:**

The aim of the study was to evaluate the associations between long-term exposure to air pollution and nonaccidental and cause-specific mortality in the Netherlands based on existing national databases.

**Methods:**

We used existing Dutch national databases on mortality, individual characteristics, residence history, neighborhood characteristics, and national air pollution maps based on land use regression (LUR) techniques for particulates with an aerodynamic diameter ≤ 10 μm (PM_10_) and nitrogen dioxide (NO_2_). Using these databases, we established a cohort of 7.1 million individuals ≥ 30 years of age. We followed the cohort for 7 years (2004–2011). We applied Cox proportional hazard models adjusting for potential individual and area-specific confounders.

**Results:**

After adjustment for individual and area-specific confounders, for each 10-μg/m^3^ increase, PM_10_ and NO_2_ were associated with nonaccidental mortality [hazard ratio (HR) = 1.08; 95% CI: 1.07, 1.09 and HR = 1.03; 95% CI: 1.02, 1.03, respectively], respiratory mortality (HR = 1.13; 95% CI: 1.10, 1.17 and HR = 1.02; 95% CI: 1.01, 1.03, respectively), and lung cancer mortality (HR = 1.26; 95% CI: 1.21, 1.30 and HR = 1.10 95% CI: 1.09, 1.11, respectively). Furthermore, PM_10_ was associated with circulatory disease mortality (HR = 1.06; 95% CI: 1.04, 1.08), but NO_2_ was not (HR = 1.00; 95% CI: 0.99, 1.01). PM_10_ associations were robust to adjustment for NO_2_; NO_2_ associations remained for nonaccidental mortality and lung cancer mortality after adjustment for PM_10_.

**Conclusions:**

Long-term exposure to PM_10_ and NO_2_ was associated with nonaccidental and cause-specific mortality in the Dutch population of ≥ 30 years of age.

**Citation:**

Fischer PH, Marra M, Ameling CB, Hoek G, Beelen R, de Hoogh K, Breugelmans O, Kruize H, Janssen NA, Houthuijs D. 2015. Air pollution and mortality in seven million adults: the Dutch Environmental Longitudinal Study (DUELS). Environ Health Perspect 123:697–704; http://dx.doi.org/10.1289/ehp.1408254

## Introduction

Long-term exposure to air pollution has been associated with mortality in several cohort studies (Abbey et al. 1999; Beelen et al. 2014; Brunekreef et al. 2009; Carey et al. 2013; Cesaroni et al. 2013; Chen et al. 2013; Crouse et al. 2012; Dockery et al. 1993; Hales et al. 2012; Huss et al. 2010; Pope et al. 1995; Yap et al. 2012; Zeger et al. 2008). Although the evidence is increasing, heterogeneity in size of effect estimates between cohort studies has been identified (Hoek et al. 2013).

Cohort studies specifically designed for investigating individual risk factors are time consuming, labor intensive, often limited in size, and relatively costly. To overcome these disadvantages, recent studies have linked existing national databases of air pollution, nonaccidental mortality, individual characteristics, and residential history to assess the relationships between air pollution and mortality more efficiently (Carey et al. 2013; Cesaroni et al. 2013; Chen et al. 2013; Crouse et al. 2012; Hales et al. 2012; Huss et al. 2010; Zeger et al. 2008). The aim of our study was to use existing national databases to evaluate the associations of long term exposure to air pollution [particulates with an aerodynamic diameter ≤ 10 μm (PM_10_) and nitrogen dioxide (NO_2_)] with nonaccidental and cause-specific mortality in a cohort of 7.1 million Dutch residents.

## Methods

*The study cohort*. In the Netherlands, population statistics are compiled by Statistics Netherlands (http://www.cbs.nl/en-GB/menu/home/default.htm) and are based on digital municipal population registers (Prins 2000). This registration system is known as the GBA (Gemeentelijke Basis Administratie), the municipal basic registration of population data. The GBA was implemented on 1 October 1994.

Statistics Netherlands combines the data from the GBA into a longitudinal file for each individual registered in the GBA (de Bruin et al. 2004). These records start on 1 January 1995. Changes in demographic attributes (e.g., death, address, marital status, emigration) are updated yearly by adding additional information on the nature and the date of the change. In these files, the individual identification number of the GBA is replaced by a meaningless, but unique, identification number. This identification number is used to enrich the individual files with information from other central data sources maintained by Statistics Netherlands such as the social statistical database, which contains, among others, data from the tax authorities and about employment status (Arts and Hoogteijling 2002).

From the database with the longitudinal files of all Dutch inhabitants, we selected all individuals of ≥ 30 years of age on 1 January 2004, living at the same residential address since 1 January 1999. We used data about sex, age, marital status, and region of origin. The data about origin distinguishes between Dutch, Western origin, and non-Western origin. Individuals of non-Western origin are those born in or with a parent born in Africa, Asia (except Japan and Indonesia, who are categorized as “Western origin”), or Latin America. Given the relatively large size of groups within non-Western origin, a distinction is made among those with Turkey, Morocco, and Suriname origin. Furthermore, we enriched the database with standardized disposable household income. This individual socioeconomic indicator is adjusted for differences in household size and composition.

We also used a socioeconomic indicator at four-digit postal code level. These postal code areas comprise on average about 4,000 inhabitants. This social status indicator is derived every 4 years by the Netherlands Institute for Social Research (http://www.scp.nl/english/) (Knol 1998). Each postal code area receives a unique ranking for social status according to the income level, unemployment rate, and education level of its inhabitants. The ranking is transformed to a 0–1 scale, with 1 being the lowest possible ranking on social status within the Netherlands. We took the indicator from 2002 and linked it to the cohort through the postal code of the residential addresses.

The follow-up period of the cohort was from 1 January 2004 to 1 January 2011. Subjects were lost to follow-up if their final record in the longitudinal file ended before 1 January 2011 and death was not registered as a reason for termination. Emigration was the main cause of censoring.

The Dutch population registers are intended primarily for municipal administrative purposes. However, many national and nongovernmental organizations benefit from them as well. Given the confidential character of the data, there is no free access to the population registers. Each organization interested in receiving data on a regular basis is given the opportunity to use the data upon request to the Ministry for the Interior. The Ministry decides to which data the organization gets access.

All our analyses were performed within strict privacy rules; that is, only researchers who received a signed permit were allowed to do analyses within a secured environment at our Institute. Before publication, Statistics Netherlands made sure that none of the analysis results showed potential reducibility to the individual level.

*Mortality outcomes*. A database with mortality data was available from Statistics Netherlands (Harteloh et al. 2010). We selected nonaccidental mortality [*International Classification of Diseases, 10th Revision* (ICD-10) codes A00-R99], circulatory disease mortality (ICD-10 codes I00-I99), respiratory disease mortality (ICD-10 codes J00-J99), and lung cancer mortality (ICD-10 codes C33–C34). A study of cause-of-death coding showed high reliability for these specific causes (> 90% for major causes of death such as cancers and acute myocardial infarction and about 85% for respiratory disease mortality) (Harteloh et al. 2010).

*Air pollution exposure assessment*. We made use of previously published land use regression (LUR) models to produce high-resolution air pollution maps (100 m × 100 m grids) of annual mean concentrations of PM_10_ and NO_2_ in 2001. Details of development and validation of the LUR models are presented elsewhere (Vienneau et al. 2010). Briefly, for both pollutants regression models were derived from annual mean concentrations for the year 2001 based on routine measurement data from the Dutch National Air Quality Monitoring Network (van Elzakker and Buijsman 2014). Predictor variables used for the modeling were traffic, land use, and topography integrated in a geographical information system. Addresses at baseline were linked to the estimated PM_10_ and NO_2_ concentration in the corresponding grid.

We did not assign PM_2.5_ (PM with diameter ≤ 2.5 μm) concentrations to cohort addresses, because in 2001 PM_2.5_ was not measured in the national monitoring network. Two monitoring studies (Cyrys et al. 2003; Eeftens et al. 2012) showed that the spatial variation of PM_10_ in the Netherlands is largely driven by PM_2.5_ (*R*^2^ = 0.76 and 0.72) with a median ratio between PM_2.5_ and PM_10_ of 0.66 that was stable over time from 2000 to 2009.

*Statistical analyses*. Statistical analyses were performed with SAS version 9.1 (SAS Institute Inc., Cary, NC, USA). We applied age-stratified Cox proportional hazards regression models to estimate the associations [hazard ratio (HR) and 95% confidence interval (CI)] between (cause-specific) mortality and long-term exposure to PM_10_ or NO_2_. We used 1-year age strata. We analyzed the data with *a*) models adjusting for age and sex (“unadjusted” model); *b*) models adjusted for age, sex, marital status, region of origin, and household income (individual confounder model); *c*) the individual confounder models extended with a socioeconomic status indicator of postal code areas (full model). In addition, we *d*) extended the full model with the second pollutant to analyze the robustness of the one-pollutant estimate when adjusted for the second pollutant. Statistical significance was defined as *p*-values < 0.05.

We explored nonlinearity in the relationships between PM_10_ and NO_2_ exposure and mortality with natural splines (2 degrees of freedom). We used the likelihood ratio test (LRT) (*p* < 0.05) to compare spline models with linear models. We analyzed these models with R version 2.15.1 (R Core Team 2014).

To assess the sensitivity of our relative risk estimates to missing individual lifestyle factor data, we assessed the association between our air pollution exposure estimates and lifestyle factors in a separate survey of adults across the Netherlands. We obtained data from health surveys from Community Health Services (GGD GHOR Nederland 2014) conducted in 2003–2005. We included data from 11 Community Health Services with available information on self-reported four-digit postal code, age, sex, marital status, level of education, region of origin, smoking, body mass index (BMI), alcohol consumption, and exercise. Criteria for alcohol consumption were defined by a national working group of experts for the purpose of the Community Health Services health surveys. We calculated the age- and sex-adjusted mean PM_10_ and NO_2_ concentrations at four-digit postal code level for different categories of smoking (current smoker, former smoker, never smoker), BMI (< 18.5, 18.5–25, 25–30, > 30), alcohol consumption (different categories of compliance to three criteria for responsible alcohol use), and exercise (compliance to 30 min of moderate exercise per day on at least 5 days per week). Subsequently, we additionally adjusted for the individual and neighborhood confounders that were also included in the Cox proportional hazard regression models [i.e., marital status, region of origin, individual socioeconomic status (using level of education in place of standardized household income, which was not available), and social status]. In addition to these regression analyses, we calculated the prevalence of the different variables under study for different categories (deciles) of PM_10_ and NO_2_ exposure.

Furthermore, in the full population we additionally adjusted for area-level smoking-related mortality estimated based upon observed lung cancer rates (Janssen and Spriensma 2012).

In addition we assessed effect modification by stratifying our analyses by sex, age (30–65 or > 65 years), socioeconomic status (five categories), and degree of urbanization (five categories). Results are graphically presented.

## Results

On 1 January 2004 the total population of the Netherlands was 16,260,465, of whom 9,936,994 had not moved residence in the previous 5 years (61%). Of these, 7,218,363 were of ≥ 30 years of age (73%) and entered the cohort. Table 1 shows the characteristics of the cohort members on 1 January 2004. During the follow-up period until 1 January 2011, 668,206 (9.3%) cohort members died from natural causes. Of these, 209,940 (31.4%) died from diseases of the circulatory system, 65,132 (9.7%) died from diseases of the respiratory system, and 53,735 (8.0%) died from lung cancer (Table 1).

**Table 1:** Characteristics of the cohort (*n* = 7,218,363) at baseline (2004).

Characteristic/category	*n* (%)
Sex
Male	3,444,166 (47.7)
Age (years)
30–40	1,053,506 (14.6)
40–50	1,765,274 (24.5)
50–60	1,804,214 (25.0)
60–70	1,256,075 (17.4)
70–80	877,444 (12.2)
≥ 80	461,850 (6.4)
Marital status
Married	4,977,475 (69.0)
Single	924,165 (12.8)
Widowed	639,451 (8.9)
Divorced	528,753 (7.3)
Other	148,519 (2.1)
Origin^*a*^
Morocco	67,444 (0.9)
Turkey	84,773 (1.2)
Suriname	91,588 (1.3)
Non-Western	119,538 (1.7)
Western	638,103 (8.8)
Dutch	6,216,917 (86.1)
Mortality (ICD-10 code)
Total nonaccidental (A00–R99)	668,206 (9.3)
Diseases of the circulatory system (I00–I99)	209,940 (2.9)
Diseases of the respiratory system (J00–J99)	65,132 (0.9)
Lung cancer (C33–C34)	53,735 (0.7)
^***a***^Individuals of non-Western origin are those born in or with a parent born in Africa, Asia (except Japan and Indonesia, categorized as “Western origin”), or Latin America. Given the relative large size of groups within non-Western origin a distinction is made between Turkey, Morocco, and Suriname origin.	

**Figure 1:**
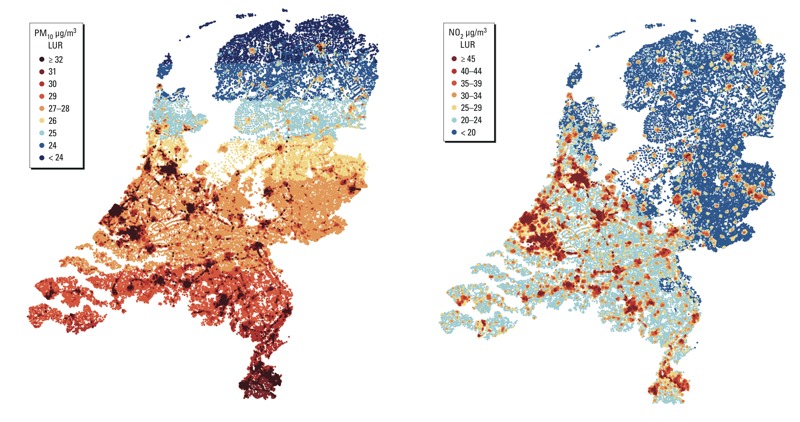
Distributions of the estimated PM_10_ and NO_2_ concentrations in the Netherlands for the year 2001 (modeled with land use regression models).

Figure 1 shows maps of the distributions of the estimated PM_10_ and NO_2_ concentrations in the Netherlands for 2001. For the addresses of the cohort members the median PM_10_ concentration was 29 μg/m^3^ [5th–95th percentile, 24 μg/m^3^–32 μg/m^3^; interquartile range (IQR) = 2.4]; the median NO_2_ concentration was 31 μg/m^3^ (5th–95th percentile, 19 μg/m^3^–44 μg/m^3^; IQR = 10.0 μg/m^3^). We estimated HRs per 10-μg/m^3^ increase in the pollutant concentration. When expressed per IQR, the estimates for PM_10_ become smaller because the IQR for PM_10_ is smaller than for NO_2_. The range (and IQR) in NO_2_ concentrations is larger than the range in PM_10_ concentrations, because NO_2_ is more influenced by local (traffic) emissions than PM_10_, which is more affected by long-range transport.

The correlation (*R*) between PM_10_ and NO_2_ was 0.58.

The results of the Cox proportional hazard analyses are presented in Tables 2 (PM_10_) and 3 (NO_2_). The highest HRs were found for both pollutants and for each category of cause of death for the “unadjusted” model. Adding individual confounders and area level for socioeconomic status reduced the magnitude of the associations.

**Table 2:** Hazard ratios (95% CIs) per 10-μg/m^3^ increase in PM_10_ levels at the home address in 2001 for nonaccidental mortality, mortality from circulatory diseases, mortality from respiratory diseases, and lung cancer mortality.

PM_10_	Nonaccidental	Circulatory diseases	Respiratory diseases	Lung cancer
Unadjusted model	1.13 (1.12, 1.14)	1.11 (1.09, 1.13)	1.18 (1.14, 1.22)	1.30 (1.25, 1.35)
Individual confounders model	1.10 (1.09, 1.11)	1.08 (1.06, 1.10)	1.16 (1.12, 1.20)	1.30 (1.25, 1.35)
Full model	1.08 (1.07, 1.09)	1.06 (1.04, 1.08)	1.13 (1.10, 1.17)	1.26 (1.21, 1.30)
Two-pollutant model	1.04 (1.03, 1.06)	1.09 (1.07, 1.12)	1.16 (1.11, 1.20)	1.09 (1.04, 1.14)
Unadjusted model: adjusted for age and sex. Individual confounders model: adjusted for age, sex, marital status, region of origin, and standardized household income. Full model: adjusted for neighborhood (postal digit) social status, in addition to the individual confounders. Two-pollutant model: adjusted for estimated NO_2_/PM_10_ at the home address in 2001, in addition to the full model covariates.				

We estimated the following associations for a 10-μg/m^3^ increase in exposure to PM_10_ and NO_2_, respectively, based on the full models: for nonaccidental mortality, HR = 1.08 (95% CI: 1.07, 1.09) and HR = 1.03 (95% CI: 1.02, 1.03); for mortality from respiratory diseases, HR = 1.13 (95% CI: 1.10, 1.17) and HR = 1.02 (95% CI: 1.01, 1.03); and for lung cancer mortality, HR = 1.26 (95% CI: 1.21, 1.30) and HR = 1.10 (95% CI: 1.09, 1.11). Only PM_10_ was associated with circulatory disease mortality (HR = 1.06; 95% CI: 1.04, 1.08 compared with HR = 1.00; 95%: 0.99, 1.01 for NO_2_)_._ For both pollutants, associations were strongest for lung cancer mortality (PM_10_ HR = 1.26; 95% CI: 1.21, 1.30; NO_2_ HR = 1.10; 95% CI: 1.09, 1.11).

In the two-pollutant models, HRs for PM_10_ decreased for nonaccidental mortality and lung cancer mortality, and increased for circulatory or respiratory disease mortality, still remaining statistically significant (Table 2). All HRs for NO_2_ decreased after adjustment for PM_10_, and the association with respiratory diseases was negative and no longer significant (Table 3).

**Table 3:** Hazard ratios (95% CIs) per 10-μg/m^3^ increase in NO_2_ levels at the home address in 2001 for nonaccidental mortality, mortality from circulatory diseases, mortality from respiratory diseases, and lung cancer mortality.

NO_2_	Nonaccidental	Circulatory diseases	Respiratory diseases	Lung cancer
Unadjusted model	1.06 (1.06, 1.06)	1.03 (1.03, 1.04)	1.05 (1.04, 1.06)	1.14 (1.13, 1.15)
Individual confounders model	1.04 (1.04, 1.04)	1.01 (1.01, 1.02)	1.03 (1.02, 1.04)	1.12 (1.11, 1.14)
Full model	1.03 (1.02, 1.03)	1.00 (0.99, 1.01)	1.02 (1.01, 1.03)	1.10 (1.09, 1.11)
Two-pollutant model	1.02 (1.02, 1.02)	0.98 (0.98, 0.99)	0.99 (0.98, 1.00)	1.08 (1.07, 1.10)
Unadjusted model: adjusted for age and sex. Individual confounders model: adjusted for age, sex, marital status, region of origin, and standardized household income. Full model: adjusted for neighborhood (postal digit) social status, in addition to the individual confounders. Two-pollutant model: adjusted for estimated NO_2_/PM_10_ at the home address in 2001, in addition to the full model covariates.				

To compare results with previous cohort studies, we calculated HRs for PM_2.5_ assuming that the association with PM_10_ is driven by the PM_2.5_ fraction (Beelen et al. 2014; Hoek et al. 2013). Based on a PM_2.5_/PM_10_ ratio of 0.66 (Cyrys et al. 2003; Eeftens et al. 2012) and PM_10_ HRs from the fully adjusted model, we estimated the following for each 10-μg/m^3^ increase in PM_2.5_: for nonaccidental mortality, HR = 1.13 (95% CI: 1.11, 1.14), for circulatory disease mortality HR = 1.09 (95% CI: 1.06, 1.12), for respiratory disease mortality HR = 1.18 (95% CI: 1.15, 1.27), and for lung cancer mortality HR = 1.41 (95% CI: 1.34, 1.49).

**Figure 2:**
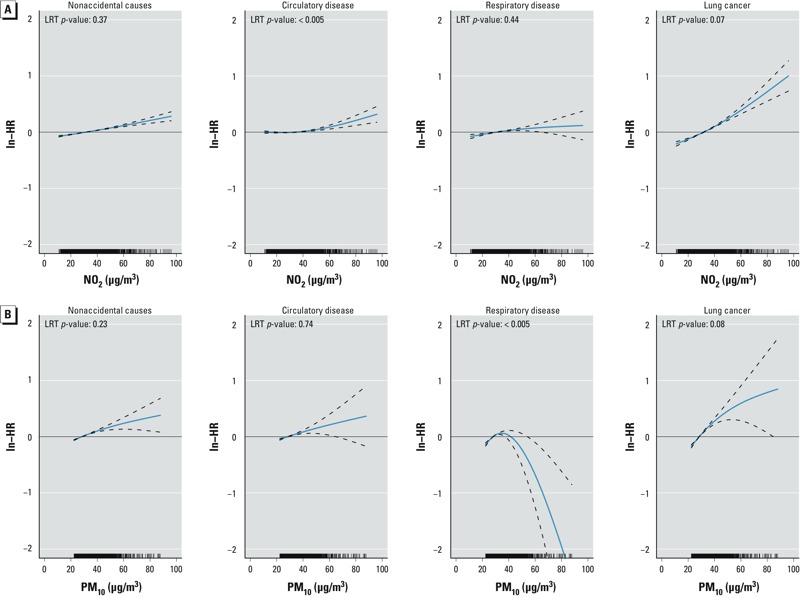
Estimated concentration–response curves (solid blue lines) and 95% CIs (dashed lines) for nonaccidental mortality, circulatory disease mortality, respiratory disease mortality, and lung cancer mortality for (*A*) NO_2_ and (*B*) PM_10_. ln, natural logarithm. Model was adjusted for age, sex, marital status, region of origin, and household income.

Associations between PM_10_ and nonaccidental, circulatory disease and lung cancer mortality did not deviate significantly (*p* < 0.01) from linear (Figure 2). The association with respiratory disease mortality increased up to about 40 μg/m^3^ and then unexpectedly decreased. Associations between NO_2_ and mortality deviated significantly from linear only for circulatory disease (Figure 2).

In addition to our main analysis, we conducted a separate analysis of associations of PM_10_ and NO_2_ with potential individual- and group-level confounders that were not available for the nationwide study sample, because the presence or absence of associations would help clarify the likelihood of confounding by these characteristics. After excluding data from adults < 30 years of age, information on lifestyle factors from 63,796 subjects was available, from 1,517 of the 3,985 four-digit postal code areas across the Netherlands. Age- and sex-adjusted mean PM_10_ and NO_2_ for different categories of selected sociodemographic characteristics and lifestyle factors are presented in the Supplemental Material, Tables S1 and S2, respectively. PM_10_ concentrations differed statistically significantly between the various categories, although these differences were small (< 0.2 μg/m^3^). After additional adjustment for marital status, region of origin, level of education, and neighborhood social status, these differences decreased to 0.07 μg/m^3^ for smoking and 0.10 μg/m^3^ for BMI, and 95% CIs overlapped (see Supplemental Material, Table S3). For NO_2_, these differences in age- and sex-adjusted mean concentrations were somewhat larger: 0.64 μg/m^3^ for current smokers compared with never smokers and 0.80 for BMI > 30 compared with 18.5 < BMI < 25. After additional adjustment for marital status, region of origin, level of education, and neighborhood social status, these differences decreased to 0.46 μg/m^3^ both for smoking and for BMI, but remained statistically significant (see Supplemental Material, Table S3). When we evaluated differences in prevalence of lifestyle and sociodemographic characteristics across different categories (deciles) of PM_10_ and NO_2_ exposure, the percentage of current smokers and participants with BMI > 30 are highest in the highest two deciles of PM_10_ and NO_2_ exposure. However, the percentage of non-Dutch nationality, low education, and average neighborhood social status score follow a similar pattern, except for non-Dutch nationality in the highest decile of PM_10_ (see Supplemental Material, Table S4).

Additional adjustment for smoking-attributable mortality reduced the HR for PM_10_ by about 30% for non-accidental mortality (from 1.081 to 1.058) to 40% for the cause-specific mortalities (see Supplemental Material, Table S5). The HRs for NO_2_ changed marginally for nonaccidental mortality (from 1.027 to 1.022), for deaths from diseases of the circulatory system (nonsignificant), and for lung cancer mortality (from 1.097 to 1.083). The NO_2_ HR for respiratory mortality changed from 1.015 to a nonsignificant 1.003.

**Figure 3:**
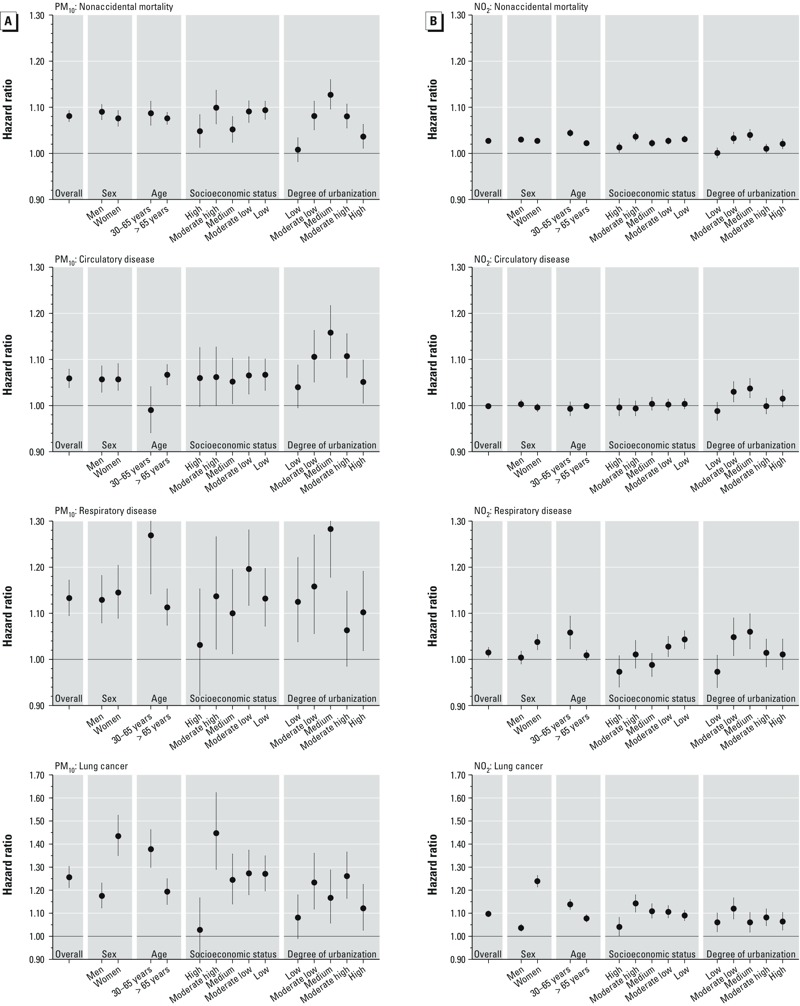
Adjusted hazard ratios (95% CIs) per 10-μg/m^3^ increase in PM_10_ (*A*) and NO_2_ (*B*), by population characteristics and cause of death. Model was adjusted for age, sex, marital status, region of origin, socioeconomic status, and household income, stratified as indicated on *x*-axis. Socioeconomic status categories are based on the quintiles of the social status rankings; urbanization is based on address density: (low: < 500 addresses/km^2^; moderate low: 500–1,000 addresses/km^2^; medium: 1,000–1,500 addresses/km^2^; moderate high: 1,500–2,500 addresses/km^2^; high: > 2,500 addresses/km^2^).

In Figure 3 we present the HRs for nonaccidental, circulatory, respiratory, and lung cancer mortality per 10 μg/m^3^ PM_10_ and NO_2_ by sex, age, social economic status and degree of urbanization. We did not find consistent patterns of effect modification across the different outcomes, although, except for circulatory mortality, HRs tended to be closer to unity among those > 65 years of age compared with younger residents. For lung cancer, women were at higher risks for both, PM_10_ and NO_2_ exposure.

## Discussion

In this large Dutch nationwide population cohort of > 7 million adults we observed positive significant associations between estimated long-term exposure to air pollution (PM_10_ and NO_2_) at the home address and nonaccidental, circulatory disease, respiratory disease, and lung cancer mortality. We used large national demographic and geographical databases to assess these associations. Our results suggest that PM_10_ is more consistently associated with mortality than NO_2_.

The large size of the cohort allowed us to assess the small relative risks of ambient air pollution with more precision than in typical individual cohort studies. Interestingly, we found highly significant associations between PM_10_ and respiratory mortality. Associations between long-term exposure to fine particles and respiratory mortality have been inconsistent in previous individual cohort studies, partly due to a relatively small number of cases (Hoek et al. 2013). Our findings are in agreement with time-series studies based on registries that have shown consistent associations between day-to-day variation in air pollution and respiratory mortality. Consistent with the time-series studies, our estimates for respiratory mortality are larger than for nonaccidental mortality.

Another interesting finding is that although circulatory disease mortality was significantly associated with PM_10_, hazard ratios were smaller than for nonaccidental mortality. Overall, in previous cohort studies, hazard ratios were larger for circulatory disease mortality, though with considerable variation between studies (Hoek et al. 2013). We speculate that better medication has reduced cardiovascular mortality, complicating assessment of associations with risk factors including air pollution.

Recently, several studies were published that were based on national demographic databases linked to air pollution data (Carey et al. 2013; Cesaroni et al. 2013; Chen et al. 2013; Crouse et al. 2012; Hales et al. 2012; Huss et al. 2010; Zeger et al. 2008). Three systematic reviews on the health effects of long-term exposure to air pollution (Brook et al. 2010; Chen et al. 2008; Hoek et al. 2013) give an extensive overview of the published literature on mortality and long-term exposure to air pollution. In Supplemental Material, Table S6, we summarize the results of the national registry studies and recent papers on mortality outcomes from a large European study on air pollution and health. For comparisons, we added our results into the table.

In our study we found particulate matter to be associated with all outcome measures that we have analyzed. Our relative risk estimate for PM_10_ on total mortality is higher than the relative risk estimate from a recent published study based on 19 European cohorts (Beelen et al. 2014). Only two other registry-based studies assessed the associations between mortality and PM_10_ (Carey et al. 2013; Hales et al. 2012). Both studies, like ours, reported higher HRs for respiratory mortality than for cardiovascular mortality (although the Hales study included lungcancer mortality). A Dutch study by Beelen et al. (2008) also estimated higher HRs for respiratory mortality than for cardiovascular mortality (for PM_2.5_), and Cesaroni et al. (2013) in Rome, Italy, reported higher HR for PM_2.5_ for cardiovascular mortality than for respiratory mortality. For NO_2_ we found statistically significant associations with all outcomes except for circulatory disease mortality, which is in line with earlier Dutch cohort study results (Beelen et al. 2008) in which a nonsignificant association for NO_2_ was reported. Again, our estimate on total mortality is higher than the relative risk estimate from the recent published European study (Beelen et al. 2014). Our results for nonaccidental mortality are comparable with the results from the registry studies by Carey et al. (2013) and Cesaroni et al. (2013) and the Dutch cohort study by Beelen et al. (2008). Cause-specific results show more heterogeneity between the different studies presented in Supplemental Material, Table S6. In the analyses in which we adjusted associations with NO_2_ for PM_10_ concentrations, the association between NO_2_ and respiratory mortality disappeared while the PM_10_ effects remained. This suggests that mortality effects of particles are not explained exclusively by traffic, as was suggested in a recent European study as well (Beelen et al. 2014).

Our estimates for lung cancer mortality for PM_10_ and NO_2_ are higher than those published recently in the national cohorts (Carey et al. 2013; Cesaroni et al. 2013; Hales et al. 2012; Huss et al. 2010), but comparable with the PM_10_ estimate for lung cancer incidence in a recently published study on the relation between long term exposure to air pollution and lung cancer incidence in 14 European cohorts (Raaschou-Nielsen et al. 2013). Adjustment for NO_2_ levels reduced the estimate (HR = 1.093; 95% CI: 1.044, 1.144), whereas the estimate for NO_2_ was HR = 1.080 (95% CI: 1.065, 1.096). This suggests that PM_10_ and NO_2_ represent different characteristics of the air pollution mixture that act independently, which may be related to the source of the pollution variability. In the European study, no association with NO_2_ was reported (Raaschou-Nielsen et al. 2013).

We found a tendency for lower HRs in the older age category (> 65 years). This is in line with the suggestion of effect modification by age in the Rome cohort (Cesaroni et al. 2013) and consistent with a previous study in Norway (Naess et al. 2007). In contrast with Cesaroni et al. (2013), we did not estimate stronger associations for men than women; and for lung cancer, associations with both exposures were significantly stronger for women than men. We did not find clear evidence of modification by socioeconomic status, which is in line with the findings in the Rome cohort.

Although, in general, studies of long-term effects of air pollution on mortality qualitatively show similar results, differences in the quantitative outcomes remain.

Our results contribute to the evidence linking long-term ambient air pollution exposure to increased nonaccidental and cause-specific mortality. Our study has several strengths. The study size is very large and includes all Dutch citizens of ≥ 30 years of age in 2004, living at least for 5 years at the 2004 address, which improves the long-term exposure classification. We used address level for all cohort members, based on the annual mean NO_2_ and PM_10_ concentrations at a 100 m × 100 m grid. Further, because of the relatively low correlation between the two air pollutant components (*R* = 0.58), we were able to disentangle the relative importance of the two components when adjusting for each other. We had individual information about important predictors of mortality at both the individual and ecological levels.

Apart from the strengths, there are also limitations of our study. Exposures were estimated by a LUR model for the year 2001 and assigned to the follow-up period 2004–2011. Although the exposure assignment precedes the follow-up period, we are not sure that the 2001 annual average adequately represents a longer exposure window, which is relevant for long-term exposure. However, there is evidence from the literature that spatial distribution of air pollution is stable over 10-year periods (Cesaroni et al. 2012; Eeftens et al. 2011; Gulliver et al. 2011). Still, people might have moved since 2004 to unknown addresses and therefore changed their exposure.

Because of the limited availability of individual lifestyle factors, we adjusted for individual and area-level SES, as in the American Cancer Society study (ACS) (Krewski et al. 2009) and the European Study of Cohorts for Air Pollution Effects (ESCAPE) (Beelen et al. 2014). The two confounders represent different “contextual” environments. Overadjustment is a possibility, but unlikely, because the correlation between the two covariates was low (–0.11).

No information on individual risk factors such as smoking, diet, alcohol use, and obesity was available. We evaluated the possibility that uncontrolled confounding from lifestyle factors may have biased our results using data from > 60,000 30- to 65-year-old participants of health surveys conducted in 2003–2005 by 11 Community Health Services. We do not have appropriate references to substantiate the representativeness of this subgroup in comparison to our national cohort, but because the regions were spread over the Netherlands and the goal of the surveys is to select random samples, we think that exposure ranges are well covered in the subgroup and comparable with the exposure range in the national cohort.

After adjustment for age, sex, marital status, region of origin, level of education, and neighborhood social status score, mean PM_10_ and NO_2_ exposures were only 0.1 μg/m^3^ (PM_10_) and 0.5 μg/m^3^ (NO_2_) higher among current smokers compared with never smokers, and among participants with BMI > 30 compared to 18.5 < BMI < 25. When looking at the prevalence of lifestyle and sociodemographic characteristics across different categories (deciles), we found that these differences were likely driven by a higher percentage of current smokers and participants with high (> 30) BMI in the highest two deciles of PM_10_ and NO_2_ exposure.

As we observe associations for the whole exposure distribution, we think it is unlikely that uncontrolled confounding from smoking or BMI has substantially biased our results (Figure 2; see also Supplemental Material, Tables S1–S4.)

In a second sensitivity analysis, we used regional age-standardized smoking attributable mortality fractions, 22–30% for men and 7–14% for women, in 40 NUTS-3 (nomenclature of territorial units for statistics) regions (Janssen and Spriensma 2012). The average population in these regions is about 400,000. We found that additional adjustment in the analyses with an area-level proxy for smoking reduces the HRs but did not materially affect the conclusions of the study (see Supplemental Material, Table S5). Several cohort studies have been published with missing data on, presumably, important individual confounders (see Supplemental Material, Table S6), but most of them showed that relative risk estimates did not materially change when some additional adjustment was made by using proxies for individual risk factors on an aggregated level (Cesaroni et al. 2013; Chen et al. 2013; Villeneuve et al. 2011; Zeger et al. 2008). In general, this suggests evidence that the lack of individual data on smoking and BMI did not bias the results in such a way that conclusions drawn on data with missing information for some individual potential confounders are materially wrong. Krewski et al. (2005a, 2005b) showed that adjusting for self-reported smoking had little effects on the relative risk estimates in two different U.S. cohorts. In the National English Cohort (Carey et al. 2013) the effect estimates were robust to adjustment for individual smoking and BMI (collected from individual patient cohorts). This was also acknowledged by Hoek et al. (2013), who concluded that effect estimates from the three large population cohorts without individual smoking data (Cesaroni et al. 2013; Crouse et al. 2012; Zeger et al. 2008) were not higher than those from the individual cohort studies.

## Conclusions

Long-term exposure to particulate air pollution (PM_10_) and NO_2_ was associated with nonaccidental mortality, mortality from respiratory diseases, and lung cancer mortality in our study population of 7 million adults. Furthermore, PM_10_ was associated with cardiovascular mortality. Associations with PM_10_ were robust to adjustment for NO_2_, and associations of NO_2_ with nonaccidental and lung cancer mortality remained after adjustment for PM_10_.

## Supplemental Material

(515 KB) PDF
