# Supplementary material for: Air Pollution and Mortality in Seven Million Adults: The Dutch Environmental Longitudinal Study (DUELS)
Source: Environ Health Perspect. 2015 Mar 11;123(7):697–704. doi: 10.1289/ehp.1408254 (PMC4492265; doi:10.1289/ehp.1408254)
Supplement: (515 KB) PDF [file ehp.1408254.s001.acco.pdf]

Environ Health Perspect
doi: 10.1289/ehp.1408254

Note to Readers: ***EHP*** **strives to ensure that all journal content is accessible to all readers. However, some figures and Supplemental Material published in *EHP* articles may not conform to 508 standards due to the complexity of the information being presented. If you need assistance accessing journal content, please contact ehp508@niehs.nih.gov. Our staff will work with you to assess and meet your accessibility needs within 3 working days.**

Supplemental Material

Air Pollution and Mortality in Seven Million Adults: The Dutch Environmental Longitudinal Study (DUELS)

Paul H. Fischer, Marten Marra, Caroline B. Ameling, Gerard Hoek, Rob Beelen, Kees de Hoogh, Oscar Breugelmans, Hanneke Kruize, Nicole A.H. Janssen, and Danny Houthuijs

Table of Contents

Table S1. Age and sex adjusted mean PM_{10} and NO_2 concentrations ($\mu g/m^3$) at 4-digit postal code level for selected socio-demographic characteristics among participants of the 2003 to 2005 surveys of 11 Community Health Services (n=63,796).

Table S2. Age and sex adjusted mean PM_{10} and NO_2 concentrations ($\mu g/m^3$) at 4 digit postal code level for selected behavioral chronic disease factors among participants of the 2003 to 2005 surveys of 11 Community Health Services (n=63.796).

Table S3. Age and sex adjusted mean PM_{10} and NO_2 concentrations ($\mu g/m^3$) at 4-digit postal code level for selected behavioral chronic disease factors among participants of the 2003 to 2005 surveys of 11 Municipal Health Services. Also adjusted for marital status, region of origin[a], education, and neighborhood social status-score (n=61.121).

Table S4. Percentages per decile of PM_{10} and NO_2 concentrations ($\mu g/m^3$) at 4-digit postal code level for selected behavioral chronic disease factors among participants of the 2003 to 2005 surveys of 11 Municipal Health Services.

Table S5. Hazard ratios and 95% confidence intervals per 10 $\mu g/m^3$ increase in PM_{10} concentrations at the home address in 2001 of the whole study population for natural mortality, mortality from circulatory diseases, mortality from respiratory diseases, and lung cancer mortality.

Table S6. Summary of main cohort characteristics of registry based cohort studies, recently published European cohort studies and Dutch NLCS study.

References to sources sited in Table S6

Table S1. Age and sex adjusted mean PM_{10} and NO_2 concentrations ($\mu g/m^3$) at 4-digit postal code level for selected socio-demographic characteristics among participants of the 2003 to 2005 surveys of 11 Community Health Services (n=63,796).

Characteristic	N	Mean PM_{10}	(95% CI)	Mean NO_2	(95%CI)	
**Sex**						
Male	29,103	29.40	(29.38 - 29.42)	31.81	(31.74 -	31.88)
Female	34,693	29.33	(29.31 -29.35)	31.71	(31.66 -	31.78)
**Age**						
30-34	6,931	29.46	(29.42 -29.50)	32.23	(32.10 -	32.37)
24-39	8,911	29.40	(29.37 -29.44)	31.65	(31.54 -	31.77)
40-44	9,812	29.35	(29.32 - 29.39)	31.46	(31.35 -	31.58)
45-49	9,738	29.40	(29.36 -29.43)	31.63	(31.51 -	31.74)
50-54	9,544	29.39	(29.35 -29.42)	31.73	(31.61 -	31.84)
55-59	10,563	29.29	(29.25 -29.32)	31.93	(31.82 -	32.03)
60-65	8,297	29.28	(29.24 -29.32)	31.71	(31.59 -	31.84)
**Marital status**						
Married	47,593	29.33	(29.31 -29.34)	31.29	(31.24 -	31.35)
Living together	6,113	29.37	(29.33 -29.42)	32.36	(32.22 -	32.51)
Divorced	3,906	29.56	(29.51 -29.62)	33.88	(33.71 -	34.06)
Single	4,676	29.53	(29.49 -29.58)	33.61	(33.45 -	33.78)
Widowed	1,285	29.57	(29.48 -29.67)	31.99	(31.67 -	32.30)
missing	223					
**Origin**						
Dutch	57,297	29.36	(29.35 -29.37)	31.43	(31.38 -	31.48)
Non-Dutch, western	2,771	28.96	(28.90 -29.02)	33.15	(32.94 -	33.36)
non western	2,769	29.66	(29.59 -29.72)	36.94	(36.73 -	37.15)
missing	959					
**Education**						
Higher	15,755	29.29	(29.27 -29.33)	32.17	(32.08 -	32.26)
Intermediate	17,811	29.28	(29.25 -29.30)	31.34	(31.26 -	31.43)
Lower	23,733	29.40	(29.38 -29.42)	31.46	(31.39 -	31.53)
Primary	4,767	29.72	(29.67 -29.77)	33.25	(33.08 -	33.41)
missing	1,730					

Table S2. Age and sex adjusted mean PM_{10} and NO_2 concentrations (μg/m^3) at 4 digit postal code level for selected behavioral chronic disease factors among participants of the 2003 to 2005 surveys of 11 Community Health Services (n=63.796).

Characteristic	N	Mean PM_{10}	(95% CI)	Mean NO_2	(95%CI)
**Smoking**					
Current smoker	17,251	29.46	(29.43 - 29.48)	32.31	(32.22 - 32.39)
Former smoker	22,961	29.34	(29.32 - 29.37)	31.43	(31.35 - 31.51)
Never smoker	22,966	29.32	(29.29 - 29.34)	31.67	(31.59 - 31.74)
missing	618				
**BMI**					
BMI<18.5	649	29.60	(29.47 - 29.74)	32.55	(32.12 - 32.99)
18.5<BMI<25	30,713	29.34	(29.32 - 29.36)	31.60	(31.54 - 31.67)
25<BMI<30	23,068	29.37	(29.35 - 29.39)	31.76	(31.69 - 31.84)
BMI>30	7,506	29.52	(29.48 - 29.56)	32.40	(32.27 - 32.52)
missing	1,860				
**Exercise (complies to standard)[a]**					
Yes	30,743	28.83	(28.81 - 28.85)	31.19	(31.13 - 31.26)
No	17,649	28.87	(28.85 - 28.89)	31.71	(31.63 - 31.80)
Missing[b]	15,404				
**Alcohol use (compliance to criteria[c])**					
Does not drink	8,810	29.41	(29.37 - 29.45)	32.63	(32.51 - 32.75)
Complies to all 3 criteria	30,366	29.32	(29.30 - 29.34)	31.57	(31.50 - 31.63)
Exceeds 1-2 criteria	17,513	29.36	(29.33 - 29.38)	31.57	(31.48 - 31.66)
Exceeds all 3 criteria	2,6452	29.42	(29.36 - 29.49)	32.36	(32.14 - 32.57)
missing	4,455				

[a]Compliance to 30 minutes of moderate exercise per day on at least 5 days per week. [b]Not available for 2 of the 11 surveys. [c]Criteria for responsible alcohol use:

1. men: a maximum of 21 glasses per week; women: a maximum of 14 glasses per week
2. men: a maximum of 5 glasses per drinking day; women: a maximum of 3 glasses per drinking day
3. maximum 5 drinking days per week

Table S3. Age and sex adjusted mean PM_{10} and NO_2 concentrations (μg/m^3) at 4-digit postal code level for selected behavioral chronic disease factors among participants of the 2003 to 2005 surveys of 11 Municipal Health Services. Also adjusted for marital status, region of origin[a], education, and neighborhood social status-score (n=61.121).

Characteristic	N	Mean PM_{10}	(95% CI)	Mean NO_2	(95%CI)
**Smoking**					
Current smoker	16,454	29.45	(29.39 - 29.51)	35.91	(35.71 - 36.11)
Former smoker	22,079	29.41	(29.35 - 29.47)	35.42	(35.23 - 35.62)
Never smoker	22,043	29.38	(29.32 - 29.44)	35.45	(35.26 - 35.65)
missing	545				
**BMI**					
BMI<18.5	617	29.57	(29.43 - 29.72)	35.97	(35.51 - 36.44)
18.5<BMI<25	29,593	29.38	(29.32 - 29.44)	35.39	(35.19 - 35.58)
25<BMI<30	22,172	29.39	(29.32 - 29.45)	35.53	(35.33 - 35.73)
BMI>30	7,164	29.48	(29.41 - 29.54)	35.85	(35.63 - 36.07)
missing	1,575				
**Exercise (complies to standard)[b]**					
Yes	29,884	29.44	(29.38 - 29.50)	35.66	(35.45 - 35.88)
No	17,003	29.45	(29.39 - 29.51)	35.84	(35.62 - 36.06)
Missing[c]	14,234				
**Alcohol use (compliance to criteria[d])**					
Does not drink	8,170	29.37	(29.30 - 29.43)	35.75	(35.54 - 35.96)
Complies to all 3 criteria	29,326	29.43	(29.36 - 29.49)	35.54	(35.34 - 35.75)
Exceeds 1-2 criteria	16,981	29.47	(29.41 - 29.54)	35.58	(35.37 - 35.79)
Exceeds all 3 criteria	2,559	29.50	(29.41 - 29.59)	36.22	(35.94 - 36.51)
missing	4,085				

[a]The data about origin distinguishes between Dutch, western origin, and non-western origin. Individuals of non-western origin are those born in or with a parent born in Africa, Asia (except Japan and Indonesia, who are categorised as "western origin"), or Latin America. Given the relative large size of groups within non-western origin a distinction is made between Turkey, Morocco, and Surinam origin. [b]Compliance to 30 minutes of moderate exercise per day on at least 5 days per week. [c]Not available for 2 of the 11 surveys. [d]Criteria for responsible alcohol use:

1. men: a maximum of 21 glasses per week; women: a maximum of 14 glasses per week
2. men: a maximum of 5 glasses per drinking day; women: a maximum of 3 glasses per drinking day
3. maximum 5 drinking days per week

Table S4. Percentages per decile of PM_{10} and NO_2 concentrations ($\mu g/m^3$) at 4-digit postal code level for selected behavioral chronic disease factors among participants of the 2003 to 2005 surveys of 11 Municipal Health Services.

Exposure category	Current smoker	Former smoker	Never smoker	BMI <18.5	BMI 18.5-25	BMI 25-30	BMI>30	Dutch nationality	Low education	Mean social economic status score[a]
**PM_{10}**										
1 (<25.2 μg/m^3)	27.8	36.6	35.6	1.0	52.0	36.6	10.3	92.4	6.1	.37
2 (25.2-26.9 μg/m^3)	24.6	35.5	39.9	0.9	51.8	36.7	10.6	90.4	5.7	.31
3 (26.9-28.7 μg/m^3)	23.7	36.8	39.5	1.0	49.4	38.2	11.4	91.8	6.0	.32
4 (28.7-30.3 μg/m^3)	24.3	37.7	38.0	1.0	49.8	37.8	11.5	90.3	6.8	.36
5 (30.3-31.6 μg/m^3)	27.0	38.6	34.4	0.8	48.9	37.7	12.6	91.2	7.2	.35
6 (31.6-32.9 μg/m^3)	26.3	36.8	36.9	1.0	48.9	37.1	13.1	89.6	7.7	.35
7 (32.9-34.3 μg/m^3)	26.6	37.2	36.2	0.8	49.5	38.1	11.6	91.3	7.1	.36
8 (34.3-36.5 μg/m^3)	28.1	36.6	35.3	1.2	49.6	37.0	12.1	89.1	8.9	.37
9 (36.5-39.0 μg/m^3)	30.1	34.4	35.5	1.2	48.7	36.8	13.4	89.4	10.8	.42
10 (>39.0 μg/m^3)	34.7	33.3	32.1	1.5	47.4	36.4	14.6	96.6	10.7	.43
**NO_2**										
1 (<27.7 μg/m^3))	24.7	38.4	36.9	0.8	50.9	37.5	10.8	96.3	5.9	.37
2 (27.7-28.4 μg/m^3)	24.3	38.2	37.5	0.9	52.0	36.3	10.7	94.8	6.2	.35
3 (28.4-28.9 μg/m^3)	25.7	37.9	36.9	0.9	50.0	37.9	11.2	94.9	6.7	.35
4 (28.9-29.2 μg/m^3)	25.1	37.3	37.6	1.1	50.9	36.5	11.5	94.1	6.5	.34
5 (29.2-29.5 μg/m^3)	26.7	37.4	35.9	1.0	50.2	37.4	11.3	92.1	6.9	.33
6 (29.5-29.7 μg/m^3)	25.4	36.8	37.9	1.0	49.8	37.8	11.3	91.9	6.5	.32
7 (29.7-30.0 μg/m^3)	27.6	37.1	35.3	1.1	49.9	37.2	11.9	93.4	6.6	.35
8 (30.0-30.6 μg/m^3)	29.4	34.9	35.7	1.1	49.7	36.6	12.6	91.0	8.7	.36
9 (30.6-31.4 μg/m^3)	31.1	34.0	34.9	1.4	45.1	38.3	15.2	87.3	10.2	.41
10 (>31.4 μg/m^3)	33.1	31.4	35.5	1.2	47.4	36.7	14.6	75.9	12.9	.45

[a]Mean SES-ranking of postal code areas in exposure group (0: highest SES; 1: lowest SES).

Table S5. Hazard ratios and 95% confidence intervals per 10 μg/m^3 increase in PM_{10} concentrations at the home address in 2001 of the whole study population for natural mortality, mortality from circulatory diseases, mortality from respiratory diseases, and lung cancer mortality.

	**PM_{10}**			
**Model**	**Natural causes**	**Circulatory diseases**	**Respiratory diseases**	**Lung cancer**
Full model	1.08<br>1.07 – 1.09	1.06<br>1.04 – 1.08	1.13<br>1.10 – 1.17	1.26<br>1.21 – 1.30
Smoking adjusted model	1.06<br>1.05 – 1.07	1.03<br>1.01 – 1.06	1.08<br>1.04 – 1.12	1.16<br>1.11 – 1.20
	**NO_2**			
Full model	1.03<br>1.02 – 1.03	1.00<br>0.99 – 1.01	1.02<br>1.01 – 1.03	1.10<br>1.09 – 1.11
Smoking adjusted model	1.02<br>1.02 – 1.03	0.99<br>0.99 – 1.00	1.00<br>0.99 – 1.01	1.08<br>1.07 – 1.10

Full model: Adjusted for age, gender, marital status, region of origin, standardised household income, and neighbourhood (postcode) measure for social status (full model results correspond to the values shown in Tables 2 and 3).

Smoking adjusted model: Adjusted for age, gender, marital status, region of origin, standardised household income, neighbourhood (postcode) measure for social status (mean SES-ranking of postal code areas in exposure group (0: highest SES; 1: lowest SES)), and regional (NUTS-3) age-standardised smoking-attributable mortality.

Table S6. Summary of main cohort characteristics of registry based cohort studies, recently published European cohort studies and Dutch NLCS study.

Study	Location	Cohort	Size	Exposure	Air pollutants	Non-accidental HR (95% CI)	Cardiovascular HR (95% CI)	Respiratory HR (95% CI)	Lung Cancer HR (95% CI)
Carey et al. 2013	England	Population 40 – 89 years	830842	Dispersion models, 1 x 1 km grid	PM_{10}, $PM_{2.5}$, NO_2	1.07 (0.99, 1.16)<br>1.13 (1.00, 1.27)<br>1.02 (1.00, 1.05)	1.00 (0.91, 1.10)<br>1.00 (0.86, 1.17)<br>1.00 (0.97, 1.03)	1.29 (1.14, 1.46<br>1.57 (1.30, 1.90)<br>1.08 (1.04, 1.13)	1.03 (0.88, 1.21)<br>1.11 (0.86, 1.43)<br>1.06 (1.00, 1.11)
Cesaroni et al. 2013	Rome, Italy	Population ≥ 30 years	1265058	$PM_{2.5}$: dis-persion 1 x 1 km grid NO_2: LUR, address	$PM_{2.5}$<br>NO_2	1.04 (1.03, 1.05)<br>1.03 (1.02, 1.03)	1.06 (1.04, 1.08)<br>1.03 (1.02, 1.04)	1.03 (0.97, 1.08)<br>1.03 (1.00, 1.06)	1.05 (1.01, 1.10)<br>1.04 (1.02, 1.07)
Chen et al. 2013	Ontario, Canada	Population 35 – 85 years	205440	LUR address	NO_2		1.09 (1.05, 1.12)		
Crouse et al. 2012	Canada	Population ≥ 25 years	2145400	City mean or 10 x 10 km grids	$PM_{2.5}$	1.10 (1.05, 1.15)	1.15 (1.07, 1.24)		
Huss et al. 2010	Switzer-land	Population ≥ 30 years	4580311	Dispersion models, 200 x 200 m grids	PM_{10}		1.00 (0.99, 1.00)		1.05 (1.03, 1.06)
Hales et al. 2012	New Zealand	Population 30 – 74 years	1065645	LUR, census area, quintiles of exposure	PM_{10}	1.07 (1.04, 1.10)	1.06 (1.01, 1.12)	1.14 (1.05, 1.23) Includes lung cancer	1.16 (1.04, 1.29)
Zeger et al. 2008	USA	Medicare MCAPS ≥ 65 years	19.1 million	ZIP-code average	$PM_{2.5}$<br>Eastern<br>Central<br>Western	 1.11 (1.08, 1.13)<br>1.09 (1.05, 1.13)<br>1.00 (0.98, 1.02)			
Fischer et al. (this paper)	Nether-lands	Population ≥ 30 years	7218363	LUR, 100 x 100 m	PM_{10}<br>NO_2	1.08 (1.07, 1.09)<br>1.03 (1.02, 1.03)	1.06 (1.04, 1.08)<br>1.00 (0.99, 1.01)	1.13 (1.10, 1.17)<br>1.02 (1.01, 1.03)	1.26 (1.21, 1.30)<br>1.10 (1.09, 1.10)
Beelen et al. 2008	Nether-lands	Cohort ≥55 years	117528	LUR, address	$PM_{2.5}$<br>NO_2	1.06 (0.97, 1.16)<br>1.03 (1.00, 1.05)	1.04 (0.90, 1.21)<br>1.02 (0.98, 1.07)	1.07 (0.75, 1.52)<br>1.11 (1.00, 1.23)	1.06 (0.82, 1.38)<br>0.97 (0.90, 1.05)
Beelen et al. 2013	Europe	Cohorts	367251	LUR, address	PM_{10}<br>$PM_{2.5}$<br>NO_2	1.04 (1.00, 1.09)<br>1.07 (1.02, 1.13)<br>1.01 (0.99, 1.03)			
Raaschou-Nielsen et al. 2013	Europe	Cohorts	312944	LUR, address	PM_{10}<br>$PM_{2.5}$<br>NO_2				1.22 (1.03, 1.45)<br>1.39 (0.91, 2.13)<br>0.99 (0.93, 1.06)
Dimako-poulou et al. 2014	Europe	Cohorts	307553	LUR, address	PM_{10}<br>$PM_{2.5}$<br>NO_2			0.86 (0.67, 1.04)<br>0,79 (0.50, 1.25)<br>0.97 (0.89, 1.05)	
Beelen et al. 2014	Europe	Cohorts	367383	LUR, address	PM_{10}<br>$PM_{2.5}$<br>NO_2		1.02 (0.92, 1.14)<br>0.98 (0.82, 1.17)<br>1.01 (0.97, 1.06)		

References to sources sited in Table S6

Beelen, R., G. Hoek, P. A. van den Brandt, R. A. Goldbohm, P. Fischer, L. J. Schouten, M. Jerrett, E. Hughes, B. Armstrong and B. Brunekreef (2008). "Long-term effects of traffic-related air pollution on mortality in a Dutch cohort (NLCS-AIR study)." Environ Health Perspect **116**(2): 196-202.

Beelen, R., O. Raaschou-Nielsen, M. Stafoggia, Z. J. Andersen, G. Weinmayr, B. Hoffmann, K. Wolf, E. Samoli, P. Fischer, M. Nieuwenhuijsen, P. Vineis, W. W. Xun, K. Katsouyanni, K. Dimakopoulou, A. Oudin, B. Forsberg, L. Modig, A. S. Havulinna, T. Lanki, A. Turunen, B. Oftedal, W. Nystad, P. Nafstad, U. De Faire, N. L. Pedersen, C. G. Ostenson, L. Fratiglioni, J. Penell, M. Korek, G. Pershagen, K. T. Eriksen, K. Overvad, T. Ellermann, M. Eeftens, P. H. Peeters, K. Meliefste, M. Wang, B. Bueno-de-Mesquita, D. Sugiri, U. Kramer, J. Heinrich, K. de Hoogh, T. Key, A. Peters, R. Hampel, H. Concin, G. Nagel, A. Ineichen, E. Schaffner, N. Probst-Hensch, N. Kunzli, C. Schindler, T. Schikowski, M. Adam, H. Phuleria, A. Vilier, F. Clavel-Chapelon, C. Declercq, S. Grioni, V. Krogh, M. Y. Tsai, F. Ricceri, C. Sacerdote, C. Galassi, E. Migliore, A. Ranzi, G. Cesaroni, C. Badaloni, F. Forastiere, I. Tamayo, P. Amiano, M. Dorronsoro, M. Katsoulis, A. Trichopoulou, B. Brunekreef and G. Hoek (2014). "Effects of long-term exposure to air pollution on natural-cause mortality: an analysis of 22 European cohorts within the multicentre ESCAPE project." Lancet **383**(9919): 785-795.

Beelen, R., M. Stafoggia, O. Raaschou-Nielsen, Z. J. Andersen, W. W. Xun, K. Katsouyanni, K. Dimakopoulou, B. Brunekreef, G. Weinmayr, B. Hoffmann, K. Wolf, E. Samoli, D. Houthuijs, M. Nieuwenhuijsen, A. Oudin, B. Forsberg, D. Olsson, V. Salomaa, T. Lanki, T. Yli-Tuomi, B. Oftedal, G. Aamodt, P. Nafstad, U. De Faire, N. L. Pedersen, C. G. Ostenson, L. Fratiglioni, J. Penell, M. Korek, A. Pyko, K. T. Eriksen, A. Tjonneland, T. Becker, M. Eeftens, M. Bots, K. Meliefste, M. Wang, B. Bueno-de-Mesquita, D. Sugiri, U. Kramer, J. Heinrich, K. de Hoogh, T. Key, A. Peters, J. Cyrys, H. Concin, G. Nagel, A. Ineichen, E. Schaffner, N. Probst-Hensch, J. Dratva, R. Ducret-Stich, A. Vilier, F. Clavel-Chapelon, M. Stempfelet, S. Grioni, V. Krogh, M. Y. Tsai, A. Marcon, F. Ricceri, C. Sacerdote, C. Galassi, E. Migliore, A. Ranzi, G. Cesaroni, C. Badaloni, F. Forastiere, I. Tamayo, P. Amiano, M. Dorronsoro, M. Katsoulis, A. Trichopoulou, P. Vineis and G. Hoek (2014).

"Long-term Exposure to Air Pollution and Cardiovascular Mortality: An Analysis of 22 European Cohorts." Epidemiology **25**(3): 368-378.

Cesaroni, G., C. Badaloni, C. Gariazzo, M. Stafoggia, R. Sozzi, M. Davoli and F. Forastiere (2013). "Long-term exposure to urban air pollution and mortality in a cohort of more than a million adults in Rome." Environ Health Perspect **121**(3): 324-331.

Chen, H., M. S. Goldberg, R. T. Burnett, M. Jerrett, A. J. Wheeler and P. J. Villeneuve (2013). "Long-term exposure to traffic-related air pollution and cardiovascular mortality." Epidemiology **24**(1): 35-43.

Crouse, D. L., P. A. Peters, A. van Donkelaar, M. S. Goldberg, P. J. Villeneuve, O. Brion, S. Khan, D. O. Atari, M. Jerrett, C. A. Pope, M. Brauer, J. R. Brook, R. V. Martin, D. Stieb and R. T. Burnett (2012). "Risk of nonaccidental and cardiovascular mortality in relation to long-term exposure to low concentrations of fine particulate matter: a Canadian national-level cohort study." Environ Health Perspect **120**(5): 708-714.

Dimakopoulou, K., E. Samoli, R. Beelen, M. Stafoggia, Z. Jovanovic Andersen, B. Hoffmann, P. Fischer, M. Nieuwenhuijsen, P. Vineis, W. Xun, G. Hoek, O. Raaschou-Nielsen, A. Oudin, B. Forsberg, L. Modig, P. Jousilahti, T. Lanki, A. Turunen, B. Oftedal, P. Nafstad, P. E. Schwarze, J. Penell, L. Fratiglioni, N. Andersson, N. Pedersen, M. Korek, U. De Faire, K. Thorup Eriksen, A. Tjønneland, T. Becker, M. Wang, B. Bueno-de-Mesquita, M.-Y. Tsai, M. Eeftens, P. H. Peeters, K. Meliefste, A. Marcon, U. Krämer, T. A. J. Kuhlbusch, M. Vossoughi, T. Key, K. de Hoogh, R. Hampel, A. Peters, J. Heinrich, G. Weinmayr, H. Concin, G. Nagel, A. Ineichen, B. Jacquemin, M. Stempfelet, A. Vilier, F. Ricceri, C. Sacerdote, X. Pedeli, M. Katsoulis, A. Trichopoulou, B. Brunekreef and K. Katsouyanni (2014). "Air Pollution and Non-Malignant Respiratory Mortality in 16 Cohorts within the ESCAPE Project." American Journal of Respiratory and Critical Care Medicine.

Hales, S., T. Blakely and A. Woodward (2012). "Air pollution and mortality in New Zealand: cohort study." J Epidemiol Community Health **66**(5): 468-473.

Huss, A., A. Spoerri, M. Egger, M. Roosli and G. Swiss National Cohort Study (2010). "Aircraft noise, air pollution, and mortality from myocardial infarction." Epidemiology **21**(6): 829-836.

Raaschou-Nielsen, O., Z. J. Andersen, R. Beelen, E. Samoli, M. Stafoggia, G. Weinmayr, B. Hoffmann, P. Fischer, M. J. Nieuwenhuijsen, B. Brunekreef, W. W. Xun, K. Katsouyanni, K. Dimakopoulou, J. Sommar, B. Forsberg, L. Modig, A. Oudin, B. Oftedal, P. E. Schwarze, P. Nafstad, U. De Faire, N. L. Pedersen, C. G. Ostenson, L. Fratiglioni, J. Penell, M. Korek, G. Pershagen, K. T. Eriksen, M. Sorensen, A. Tjonneland, T. Ellermann, M. Eeftens, P. H. Peeters, K. Meliefste, M. Wang, B. Bueno-de-Mesquita, T. J. Key, K. de Hoogh, H. Concin, G. Nagel, A. Vilier, S. Grioni, V. Krogh, M. Y. Tsai, F. Ricceri, C. Sacerdote, C. Galassi, E. Migliore, A. Ranzi, G. Cesaroni, C. Badaloni, F. Forastiere, I. Tamayo, P. Amiano, M. Dorronsoro, A. Trichopoulou, C. Bamia, P. Vineis and G. Hoek (2013). "Air pollution and lung cancer incidence in 17 European cohorts: prospective analyses from the European Study of Cohorts for Air Pollution Effects (ESCAPE)." Lancet Oncol **14**(9): 813-822.

Zeger, S. L., F. Dominici, A. McDermott and J. M. Samet (2008). "Mortality in the Medicare population and chronic exposure to fine particulate air pollution in urban centers (2000-2005)." Environ Health Perspect **116**(12): 1614-1619.
